# New classification of periodontal diseases (NCPD): an application in a sub-Saharan country

**DOI:** 10.1038/s41405-021-00071-8

**Published:** 2021-04-26

**Authors:** William Ndjidda Bakari, Diabel Thiam, Ndeye Lira Mbow, Anna Samb, Mouhamadou Lamine Guirassy, Ahmad Moustapha Diallo, Abdoulaye Diouf, Adam Seck Diallo, Henri Michel Benoist

**Affiliations:** Department of Periodontology, Institute of Odontology and Stomatology, CheikhAnta Diop University, Dakar, Senegal

**Keywords:** Periodontitis, Periodontitis

## Abstract

**Purpose:**

To determine the clinical and radiological profile of periodontitis according to the 2018 NCPD, in a Dakar (Senegal) based periodontal clinic.

**Methods:**

This is a descriptive study based on patient’s records in the periodontology clinic. The study was conducted between November 2018 and February 2020 (15 months). All periodontitis cases were included in the study. Incomplete records (due to lack of radiographic workup or unusable periodontal charting) were excluded. Periodontitis diagnosis was established based on criteria used in the 2018 NCPD. Statistical analysis was carried out using SPSS version 20.0, with the significance threshold set at 0.05.

**Results:**

A total number of 517 patient records were collected during the study period but only 127 periodontitis records were complete. The mean age of participants was 46.8 ± 13.8 years and 63.8% of participants were males. The mean plaque index and bleeding on probing (BOP) were 74% ± 21.3 and 58.1% ± 25.1, respectively. The mean maximum clinical attachment loss was 8.7 mm ±2.7, with a probing depth greater than 6 mm present in 50.4% of the sample. The median number of missing teeth was 3 (interquartile range 5–1). Pathological mobility was present in 60.6% of the patients and 78.0% had occlusion problems. Bone crest defect at the most affected site was moderate in 52.8% of cases. The ratio of bone loss to age greater than one concerned 66.1% of the sample. Generalised (81.9%), Stage IV (70.1%) and grade C (69.3%) were the most encountered diagnosis. The disease severity was associated with age (*r* = 0.241; *P* < 0.001), BOP (*r* = 0.230; *P* = 0.013) and the number of teeth with pathological mobility (*r* = 0.318; *P* < 0.001).

**Conclusion:**

Patients with periodontitis in this study had advanced forms of the disease and required multidisciplinary care. Clinical hindsight is necessary to improve this classification.

## Introduction

Periodontitis is a disease-causing the progressive destruction of the tooth-supporting apparatus, characterized by a clinical attachment loss (CAL), a radiographically assessed alveolar bone loss, the presence of periodontal pockets and gingival bleeding.^1^ It is a polymicrobial disease with an inflammatory burden that can ultimately cause tooth loss in the absence of adequate treatment.^2^ Periodontal diseases have been subjected to several classifications over the years, in view to better define and circumscribe the different pathologies contained in it, providing a suitable tool for research, therapeutics and epidemiology.^3,4^ Ever since *pyorrhoeaa alveolaris* was first classified, many experts consensus meetings were held to continuously upgrade these classifications to render them compatible to the best available knowledge at the time.^5^ Therefore, a consensus conference was held in 2017 between the European (EFP: European Federation of Periodontology) and the American (AAP: American Association of Periodontology) periodontal societies to update the latest Armitage Periodontal Disease Classification of 1999.^4^ This new classification of periodontal diseases (NCPD) including; peri-implant pathologies, proposes a definition of periodontitis and periodontal health.^4^ With the current classification, which actually introduces a new approach for periodontal screening conditions, it seemed interesting to look at the profile of patients with periodontitis in our setting. Thus, the aim of this study is to determine the clinical and radiological profile of periodontitis on a Senegalese population using the 2018 classification in a Dakar based periodontal clinic.

## Methods

### Study design and settings

**Fig. 1:**
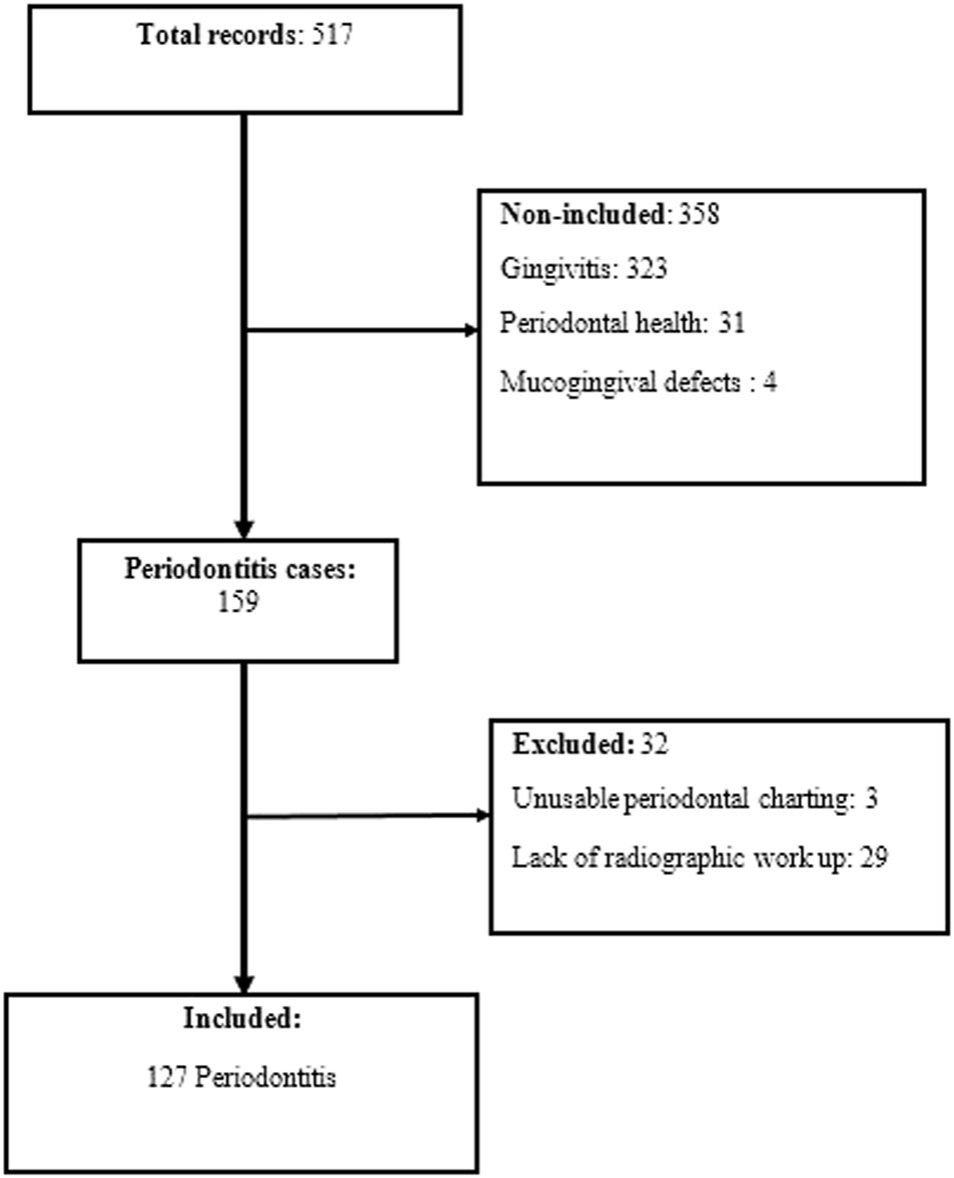
Flow chart. Showing the number of periodontitis cases during the selection process.

This was a descriptive study based on the records of patients having consulted at the periodontology clinic of the Institute of Odontology and Stomatology of the Cheikh Anta Diop University of Dakar (IOS/UCAD). Recruitment spread over the period of November 2018 to February 2020 (15 months). The records of patients who had periodontitis were included in the study. All records, whose final diagnosis was not periodontitis (periodontal health, gingivitis, muco-gingival defects, necrotizing periodontal diseases, periodontitis as manifestations of systemic disease) were not included and considered as incomplete records (lack of radiographic workup or unusable periodontal charting) were excluded (Fig. 1). At first, a minimum of 15 teeth (present in the mouth) were required in patients for consideration for a periodontal clinic. Patients were referred or invited by students as part of their periodontal clinic.

### Data collection and diagnostic criteria

The diagnosis for periodontitis in this study was established on clinical and radiological criteria used in the 2018 NCPD.^6^ The data collected were mostly on: sociodemographic variables (sex, age), risk factors (current smoking, and glycated haemoglobin levels); clinical variables including: maximum clinical attachment loss (maxCAL), probing depth (PD), tooth mobility >2 (Mühlemann et al.^7^), plaque index (PI) (O’Leary et al.^8^), bleeding on probing (BOP) (Ainamo and Bay^9^), number of missing teeth and furcation involvement (Hamp et al.^10^). Radiographic variables considered were the ratio of bone loss percentage to age, phenotype, bone resorption (horizontal, vertical, or mixed), the general level of bone destruction, the extension of alveolysis (localised <30% of teeth; generalised >30% of teeth) and bone ridge defect (moderate and severe). The Williams periodontal probe (Michigan O probe, Hu-Friedy Mfg. Co, Chicago, IL USA) was used for periodontal probing. Measurements of CAL and probing pocket depth were made at 6 (six) sites on each tooth according to the charting form used in the IOS/UCAD periodontology department. Wisdom teeth were included in the count for missing teeth. CAL and PD were estimated by the distances ranging from the cement-enamel junction (CEJ) and the free edge of the gingiva to the bottom of the sulcus. Assessment of alveolar bone lysis was made proximal to the most affected site using the technique proposed by Kornman and Papapanou^11^. Bite collapses, drifting and flaring were considered occlusion disorders. The radiographic assessment included an orthopantomogram and retro-alveolar radiographs (performed with the same apparatus for all patients) for the most affected sites identified on the orthopantomogram. Bone loss was measured by estimating the percentage distance between CEJ to the deepest part of the bone defect along the root length. Under X2 magnification with the light of the negatoscope (Fazzini negatoscope SS. Padanasuperiore, 317.20090 Vimodrome, Mi Italy), the root was divided into three parts from the CEJ to the root apex. The first part represented the coronal third, the second part the middle third and the third part the apical third.^11^ Interdental bone loss extending from the coronal third to the median third (33–50%) was considered as a moderate ridge defect. While a bone loss, exceeding 50% (from the middle third to the apical third) was regarded as a severe ridge defect. The general bone resorption level was classified as mild (coronal third), moderate (middle third) and terminal (apical third). The phenotype was assessed by seeking the proportionality between the PI found and the bone resorption observed at the most affected radiological site. There were therefore three types of phenotype: heavy biofilm deposits for a low level of bone destruction, destruction proportional to the biofilm, and destruction exceeding biofilm deposits.

The final diagnosis was made based on criteria of severity and complexity for the stage and according to the ratio of bone loss to age and risk factors for the grade (Tables 1 and 2). Two independent reviewers made the diagnosis and differences were resolved by consensus on the intervention of a third reviewer. This NCPD was the subject of several training sessions in the department to calibrate the examiners.

**Table 1:** Diagnostic criteria used for the stage of periodontitis (adapted from Tonetti et al.^6^).

Periodontitis stage		Stage I	Stage II	Stage III	Stage IV
Severity	Interdental CAL at the site of greatest loss	1–2 mm	3–4 mm	≥5 mm	≥5 mm
	Tooth loss	No tooth loss due to periodontitis		Tooth loss due to periodontitis of ≤4 dents	Tooth loss due to periodontitis of ≥5 dents
Complexity	Local	Maximum probing depth ≤4 mm. Mostly horizontal bone loss	Maximum probing depth ≤5 mm. Mostly horizontal bone loss	In addition to stage II complexity: PD ≥ 6 mm. Vertical bone loss ≥3 mm Furcation involvement class II or III Moderate ridge defect	In addition to stage III complexity: Need for complex rehabilitation due to: Masticatory dysfunction secondary occlusal trauma (tooth mobility degree ≥2) Severe ridge defect Bite collapse, drifting, flaring, less than 20 remaining teeth (10 opposing pairs)

Extent and distribution <30% of teeth involved (localised), ≥30% of teeth involved (generalized) or molar/incisor pattern.

*CAL* clinical attachment loss, *PD* probing depth.

**Table 2:** Diagnostic criteria used for the grade of periodontitis (adapted from Tonetti et al.^6^).

Periodontitis grade			Grade A: slow rate of progression	Grade B: moderate rate of progression	Grade C: rapid rate of progression
Primary criteria	Indirect evidence of progression	%Bone loss/age	<0.25	0.25 à 1.0	>1.0
		Phenotype	Heavy biofilm deposits with low levels of destruction	Destruction commensurate with biofilm deposits	Destruction exceeds expectations given biofilm deposits
Grade modifiers	Risk factors	Smoking	Non-smoker	Smoker <10 cigarettes/day	Smoker ≥10 cigarettes/day
		Diabetes (HbA1c)	Normoglycaemic/no diagnosis of diabetes	HbA1c <7.0% in patients with diabetes	HbA1c >7.0 in patients with diabetes

### Statistical analysis

Statistical analysis was carried out using SPSS (Statistical Package for Social Sciences) version 20.0. Qualitative variables were expressed as frequencies, percentages, and quantitative variables as means and standard deviations. Linear regression was used to study the relationship between the severity of the impairment designated by the maxCAL and age, PI, BOP, number of mobile teeth, and number of missing teeth. The significance threshold was set at 0.05.

## Results

A total number of 517 patient records were collected during the study period. Periodontitis was diagnosed in 159 patients, giving a prevalence of 30.8%. Only 127 periodontitis records were complete and had a radiographic record available.

**Fig. 2:**
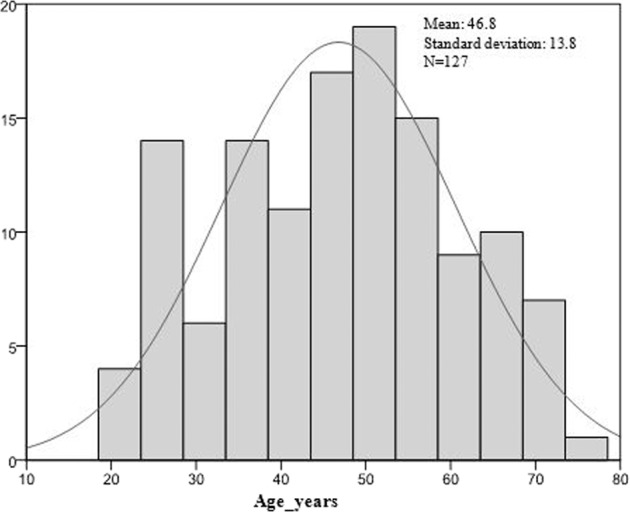
Age distribution. Showing a peak around the age of 50.

The population was predominantly male with 81 (63.8%) individuals (Table 3). The mean age was 46.8 ± 13.8 years with a range of 21–74 years. The age distribution revealed a peak in the population after 20 and 50 years old (Fig. 2). The majority of those working had jobs in the informal sector (48.0%) and 23.6% presented a periodontal risk factor of smoking and/or diabetes (Table 3).

**Table 3:** Socio-demographic characteristics.

Variables	*N* = 127	%
Sex		
Female	46	36.2
Male	81	63.8
Age (years)	Mean: 46.8 ± 13.8 min 21; max 74	
Occupation		
Jobless	38	29.9
Informal	61	48.0
Private	18	14.2
Public	10	7.9
Risk factor		
No	97	76.4
Yes	30	23.6
Smoking		
Smoker <10 cigarettes/day	9	7.1
Smoker ≥10 cigarettes/day	11	8.7
No smoking	107	84.3
Diabetes HbA1c (%)		
HbA1c > 7	17	10.2
HbA1c < 7	3	2.4
No diabetes	107	84.3

**Fig. 3:**
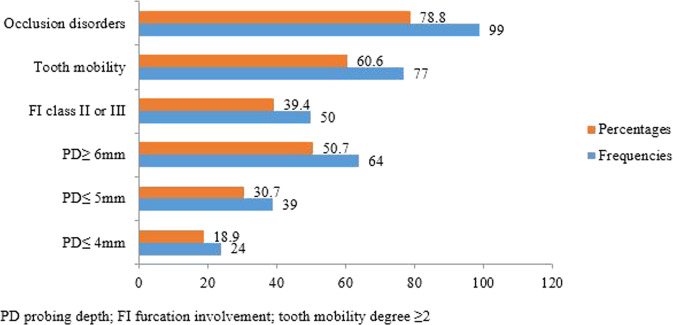
Clinical features. Showing main clinical characteristics distribution of the study population.

According to clinical data (Table 4), the mean PI and BOP were 74% ± 21.3 and 58.1% ± 25.1, respectively. The mean maxCAL was 8.7 mm ±2.7 with a range of 2–16 mm. PD greater than 6 mm (PD ≥ 6 mm) was present in 50.4% of the sample. The median number of missing teeth was 3 for a mean of 5 mobile teeth. Pathological mobility was present in 60.6% of the patients and 78.0% had occlusion problems (Fig. 3).

**Table 4:** Clinical features.

Variables	Mean	Standard deviation	Minimum	Maximum	Median
MaxCAL (mm)	8.7	2.7	2	16	8
PI (%)	74.7	21.3	13	100	78
BOP (%)	58.1	25.1	11	100	60
Missing teeth	5	4	0	17	3
Tooth mobility^a^	4	6	0	31	2

*MaxCAL* maximum interdental clinical attachment loss, *PI* plaque index O’Leary, *BOP* bleeding on probing ainamo & bay.

^a^Degree ≥2.

**Fig. 4:**
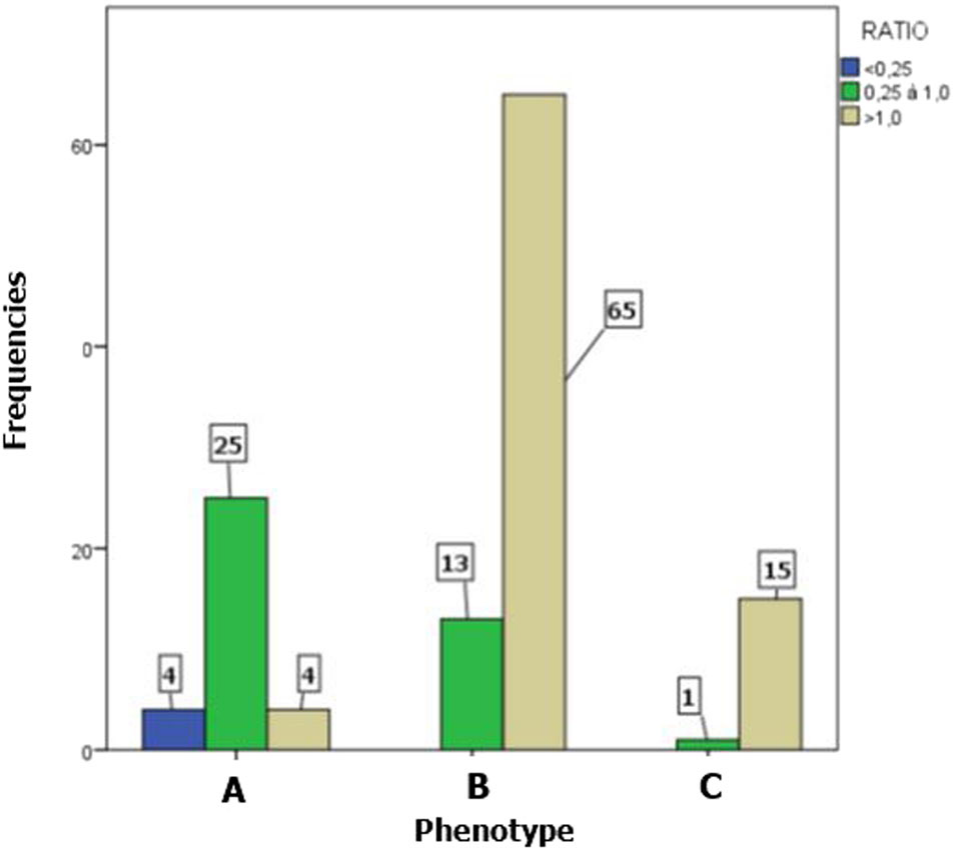
Ratio of bone loss percentage to age and phenotype. Showing a discrepancy, according to phenotype, grade B periodontitis patients have mostly a ratio greater than one.

Concerning the radiological features (Table 5), alveolysis was generalised (81.9%), mixed (66.1%) and moderate (54.3%). The ridge defect at the most affected site was moderate in 52.8% of cases. The ratio of bone loss to age greater than one (BL/age > 1) concerned 66.1% of the sample. Moreover, the phenotype was proportional in most cases (61.4%). There was a discrepancy between phenotype and age-related bone loss ratio. For a proportional phenotype, the ratio of BL/age was mostly greater than one (Fig. 4).

**Table 5:** Radiographic characteristics.

Variables	*N* = 127	%
Bone loss/age		
<0.25	4	3.1
0.25 à 1.0	39	30.7
>1.0	84	66.1
Phenotype		
Heavy biofilm/less destruction	33	26.0
Proportional	78	61.4
Light biofilm/ a lot of destruction	16	12.6
Bone loss extension		
Generalized	104	81.9
Localised	23	18.1
Alveolysis type		
Horizontal	36	28.3
Vertical	7	5.5
Mixed	84	66.1
General bone loss		
Superficial (coronal third)	12	9.4
Moderate (middle third)	69	54.3
Terminal (apical third)	46	36.2
Ridge defect		
Mild (<33%)	9	7.1
Moderate (33–50%)	67	52.8
Severe (>50%)	51	40.1

**Fig. 5:**
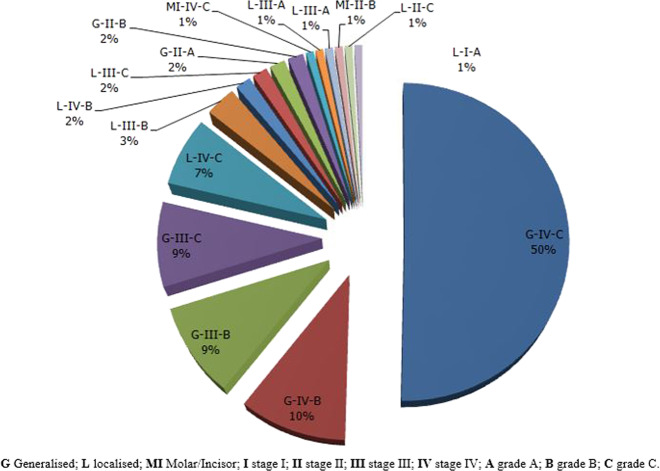
Periodontitis distribution according to clinical forms. Showing generalised stade IV grade C periodontitis as the main clinical form encountered.

Stage IV (70.1%) and grade C (69.3%) were the most encountered diagnosis. The extension was generalised in 81.9% of the sample (Table 6). Thus, the most frequently mentioned clinical form was generalised stage IV grade C periodontitis; found in 50.4% of the sample (Fig. 5). The disease severity was associated with age (*r* = 0.241; *P* < 0.001), BOP (*r* = 0.230; *P* = 0.013) and the number of teeth with pathological mobility (*r* = 0.318; *P* < 0.001) (Table 7). The maxCAL thus increased significantly with age, BOP and the number of mobile teeth.

**Table 6:** Distribution of periodontitis according to the stage, grade and extension.

Variables	*N* = 127	%
Stage		
I	2	1.6
II	6	4.7
III	31	24.4
IV	89	70.1
Grade		
A	5	3.9
B	34	26.8
C	88	69.3
Extent and distribution		
Generalized	104	81.9
Localised	21	16.5
Molar/incisor pattern	2	1.6

**Table 7:** Multiple linear regression model.

Coefficients^a^					
Model	Non-standardized coefficient	Standard error	Standardized coefficient	*t*	*P-*value
	A		Bêta		
1					
(Constant)	3.884	1.063		3.653	0.000
*AGE*	0.048	0.017	0.241	2.809	0.006^b^
*PI*	0.003	0.012	0.025	0.278	0.781
*BOP*	0.025	0.010	0.230	2.514	0.013^b^
*Mobile teeth*	0.152	0.041	0.318	3.737	0.000^b^
*Missing teeth*	0.073	0.055	0.116	1.330	0.186

^a^Dependant variable: maxCAL, t-student, PI (plaque index), and BOP (bleeding on probing).

^b^Age, BOP and number of mobile teeth are associated with clinical attachment loss.

## Discussion

This study aimed to determine the clinical and radiological profile of periodontitis according to the NCPD of 2018. Periodontitis occurs mostly in male patients, with a mean age of 47 years and those working in the informal job sector. Clinically, the patients had a mean maxCAL of 8.7 mm with PD ≥ 6 mm, a mean PI and BOP of 74% and 58% respectively, with tooth mobility degree ≥2 (Mühlemman et al.^7^), furcation grade II or III (Hamp et al.^10^) involvement and occlusion disorders. Radiographically, bone lysis was generalised, mixed with moderate ridge defects. The bone loss/age ratio was >1 for a proportional phenotype. Periodontitis was generalized stage IV and grade C.

Benoist et al.^12^ reported out of 564 patients with periodontitis, a male predominance of 53%. Whilst Ravida et al.^13^ and Graetz et al.^14^ found a female predominance of 52.1% and 60.2%, respectively, on 251 and 292 patients received in a university periodontology clinic according to the new classification. Though these studies report a higher prevalence of periodontitis in women, male predominance has been shown in the literature.^15^ However, no clear association was shown to exist between gender and the occurrence of periodontitis.^15^

The mean age was 46.8 ± 13.8 years. Benoist et al.^12^ found a mean age of 40.4 ± 14.9 years for chronic periodontitis and 28.1 ± 8.9 years for aggressive periodontitis. Chronic periodontitis represented 73.2% of their sample. Ravida et al.^13^ found a mean age of 47.3 ± 12 years while Graetz et al.^14^ found a mean age of 45.1 ± 9.6 years. These results are close to our findings confirming the trend observed. After the 1999 classification, it was acknowledged that periodontitis can occur at any age and the chronic and aggressive forms of the disease were described.^3^ In this recent classification, age plays a role in the indirect assessment of the disease progression by the bone loss to age ratio but not as a risk factor. Only diabetes and smoking remain considered as grade-modifying risk factors in the diagnosis of periodontitis.^1^ Nevertheless, age was associated with the severity of the disease in this study.

Interdental CAL is the main criterion for assessing the severity of the periodontal disease.^11,16^ The inclusion of CAL at the most affected tooth is an innovation in this classification.^6^ Clinically, our patients had a mean maxCAL of 8.7 mm indicating the severity of the disease observed. CAL in the study by Benoist et al.^12^ for chronic periodontitis was 4.4 mm representing a mean value that was not based on the most affected tooth, which explains why it is lower than that found in this study. Graetz et al.^14^ obtained a mean of 7.5 mm. The NCPD considers the maximum of the CAL, therefore values higher than those observed in studies using Armitage’s classification makes sense.^1^

Half of our participants had generalised stage IV grade C periodontitis. Ravida et al.^13^ in their study found stage III (50%) and grade B (66.1%) as the majority. Their study was retrospective, and the data recorded for the stage including information on complexity were not complete. Stage III/grade C (55.77%) was the most common in the study by Graetz et al.^14^ on 251 periodontitis cases. In Graetz’s study, risk factors were not documented, and diabetic patients were de facto classified as grade C. Moreover, the authors did not record some information like PI, mastication, and occlusion disorders. This situation suggests some bias in the classification. Since the difference between stage III and IV is mainly based on the complexity factors^1,6,16^. However, the fact that our study was conducted in a university setting, a reference care centre for the treatment of periodontal disease, may in part explain why patients showing up for consultation are generally at an advanced stage of the disease. The poverty level (46.7%) and belonging to the informal sector characterised by low daily incomes may also justify the delay in consultation and treatment. Periodontal treatment can be estimated at around US$69 in our context where the minimum income is US$106.36.^17,18^

A significant relation was observed between the severity of the damage, the BOP and tooth loss experience. Fear of causing bleeding or of further mobilizing already loose teeth during daily tooth brushing promotes deterioration of periodontal status and may explain this relation.

However, the study findings are to be interpreted within the confines of certain limitations and comments. Periodontal charting was carried out by students though crosschecked by a periodontal specialist. The periodontal aetiology of tooth loss was not verified due to the memory bias of patients who reported it.^14^ The number of missing teeth in this study was not discriminated against based on origin, which could lead to misclassification. Moreover, wisdom teeth were included in the missing teeth’s count during data analysis. The lack of consensus regarding the assessment of radiographic parameters in the literature has led to methodological choices that may be questionable: assessment of alveolysis, characterisation of ridge defect and vertical bone loss evaluation. Intra-examiner reproducibility has not been determined (intra-class correlation coefficient). Kormann’s method for assessing bone lysis leaves some grey zones in subjectivity when it comes to converting this bone loss into a percentage.^11^ Concerning severity criteria for the stage diagnosis, bone loss was not considered as a criterion, the analysis is based mainly on the maximum interdental CAL as advocated by the new classification.^6,16^ Indeed, the use of bone loss percentage is to be considered for staging in the absence of data on CAL.^1,11^ Furthermore, the most clinically affected tooth is not always the most affected on radiographic analysis, thus motivating the choice of maxCAL as the basis for diagnosis.

In the diagnosis relating to grade, the absence of a periodontal history of patients coming for consultation and the non-computerisation of medical records made it difficult to consider direct evolutionary criteria such as loss of clinical attachment or bone loss over the last 5 years.^6^ Risk factors were documented in this study. The dissociation between phenotype and percentage of bone loss raises the need for objectivity in the characterisation of the patients’ phenotype. Indeed, dental biofilm is susceptible to be removed and go to self-reconstitution, while bone loss is irreversible without therapy. The ratio of bone loss percentage to age is the main criterion for determining grade in the absence of periodontal history.^16^

## Conclusion

This NCPD brings out a new perspective on patients’ periodontal status. Periodontitis, through its characterization according to stage and grade, sees its extent and complexity considered. Periodontitis patients in this study had advanced forms of the disease with end-stage clinical features and extensive radiologic bone destruction. Clinical hindsight is necessary to adapt this classification to practice in limited-resource healthcare settings as it is in developing countries.
